# Health Care Utilization and Health Care Expenditure of Nepali Older Adults

**DOI:** 10.3389/fpubh.2019.00024

**Published:** 2019-02-15

**Authors:** Sabnam Acharya, Saruna Ghimire, Eva M. Jeffers, Naveen Shrestha

**Affiliations:** ^1^School of Health and Allied Sciences, Pokhara University, Pokhara, Nepal; ^2^Agrata Health and Education (AHEAD)-Nepal, Kathmandu, Nepal; ^3^Colorado School of Public Health, University of Northern Colorado, Greeley, CO, United States

**Keywords:** health care utilization, health care expenditure, comorbidities, Nepal, older adults

## Abstract

**Introduction:** Aging is associated with multiple chronic conditions. In older age, health needs and demand for health services utilization increase. There are limited data in Nepal on the health care utilization as well as health care costs among the elderly population. Therefore, it is imperative to explore the factors hindering access to health care among Nepalese older adults. Our study aims to explore the health care utilization and expenditure among Nepali older adults.

**Method:** A community-based cross-sectional survey was conducted among 401 older adults residing in Pokhara Lekhnath metropolitan of Nepal. The survey tool was adapted from the Study on Global Aging and Adult Health (SAGE)'s questions on “Health Care Utilization.” The predictors of health care utilization were assessed in binary logistic regression models.

**Results:** Study participants, mean (±*SD*) age 70.2 (±8.0) years, had various preexisting conditions such as hypertension (37.7 %), gastritis (28.4 %), asthma (25.4 %), and arthritis (23.4%) reported in the past 12 months but only 70% visited a health facility. A notable proportion (30%) of participants didn't utilize health services despite having a health problem. The utilization of out-patient and in-patient health services were 87.5 and 14.6% respectively. The use of private health facilities (56.4%) was high compared to the use of government health facilities (35.7%). Privileged ethnicity, living with a partner, higher annual income, knowledge of social insurance, and multi-morbidity were associated with higher odds of utilizing health services. Participants of privileged ethnicity, with higher household income, attending private health facility, and having multi-morbidities had significantly higher out of pocket health expenditures.

**Conclusions:** A notable proportion of elderly participants did not utilize health services despite having a health problem. The public health system must develop effective strategies to attract this segment of the society. High dependency on private health facilities, as noted in the study, will only lead toward higher out of pocket health expenditures. The health benefits of regular health screenings must be disseminated among the elderly population. Developing quality and affordable health care services for older adults to ensure equity in accessibility will be a major task for the public health system in Nepal.

## Introduction

The global population is aging, and so is the Nepali population (1). The population of older adults, defined by the Nepali Senior Citizens Act as individuals aged 60 years and above (2), constituted 2.1 million people in 2011 (1). The population of elderly Nepalese is burgeoning; 6.8% in 1995/96, 7.6% in 2003/04, and 9.1% in 2010/11 (1). The population growth rate of the older adult population even surpasses the nation's total population growth rate (1). Yet, knowledge regarding different aspects of geriatric health in Nepal is limited (3).

Aging is associated with multiple chronic conditions (4, 5). Increasing age is an independent risk factor for cardiovascular disease (CVD), respiratory disease, diabetes, hypertension, etc. (6–8). Chronic conditions and multi-morbidity pose a significant challenge to health care services and are associated with higher use patterns and disproportional direct health care costs (4, 5, 9). In older age, health needs, and demand for health services utilization increase (10). In the U.S., three-fourths of the total health care costs are related to the treatment of chronic conditions (9). Nepal is experiencing epidemiological, as well as demographic, transitions (11). The burden of chronic conditions and the elderly population are increasing, implying greater health care needs and appropriate provision of geriatric health services for the years to come (10).

Elderly adults are particularly vulnerable to variability in healthcare use, including both over- and under-utilization of healthcare services, which have the potential to cause unnecessary personal and financial harm (12, 13). The literature on the specific patterns of health care utilization for Nepali elderly is scarce (8, 14). However, the topic is important because healthcare utilization patterns are often complex; and wide variations in the utilization of healthcare services, unrelated to patient health outcomes, have been observed across geographic areas, providers, and payers (15, 16).

Additional factors such as poverty, illiteracy, and belief in traditional healers, further jeopardize the health needs and health services utilization of the burgeoning Nepali older adults. Nepal is a low-income country with a per capita gross domestic product of $21.13 billion and a gross national income per capita of $730 as of 2016 (17). The household expenditure on health care is only about 5.5% of the total household expenditure (18). In addition to limited financial resources, illiteracy and belief in traditional healing techniques among Nepalese may further limit their health-seeking behavior (8, 14). The literacy rate of Nepal is 65.9% (male: 75.1%; female: 57.4%) (1). More than half of Nepali women visited traditional healers for health care, and irrespective of age, gender or ethnicity, faith healers are the first choice of treatment (19, 20). To aggravate it more, the government does not have a rigorous policy to ensure availability of specialized geriatric health care for its senior citizen. Traditionally, senior citizens reside with and are taken care of by their sons and daughters-in-law (21). Sons are the primary source of health care expenditures for older people; 49% of older parents' expenses were managed by their son (11). But the traditional system of joint families has been replaced by a nuclear family, and the internal and external migration of youth leaves behind elderly parents (21). The Government of Nepal offers free health care for senior citizens at government hospitals and health centers and financial subsidies for selected diseases (21). But awareness of the free healthcare and subsidy scheme is minimal among the elderly. Despite the provision, only about half of the elderly participants reported having a check-up by a doctor in past year (21). Therefore, it is imperative to explore the factors hindering access to health care among Nepalese older adults. There is limited data in Nepal on the health care utilization as well as health care costs in the elderly population. Our study aims to explore the healthcare expenditures and the utilization of health services among the aging population living in Pokhara Lekhnath metropolitan.

## Materials and Methods

### Study Setting

A community-based cross-sectional house-to-house survey study was conducted from June to November 2017 among older adults residing in Pokhara Lekhnath metropolitan, Kaski district of Nepal. Pokhara-Lekhnath, a panoramic city located about 200 kilometers west of Nepal's capital of Kathmandu, is the largest city in Nepal, with an area of 464.24 km^2^. It is also the second most populous city with a total population of 413,934 (male: 201,107; female: 212,827). The total population of 60 years and above residing in the metropolitan was 42,935 (male: 19,536; female: 23,399) (1). The city is home to large population of Janjatis and “Gurkha” soldiers who are employed abroad and has therefore resulted in high-income levels of the locals. It is also Nepal's second most expensive city to live in and has a high literacy rate of 72.1% (1).

### Study Population

Older adults [≥60 years as defined by the Nepali Senior Citizens Act (2)] residing for a minimum of 1 year in the study area and having had any health problem in past year were eligible to participate. Mentally unstable, deaf and dumb were excluded from the study.

### Sample Size and Sampling

The sample size was calculated using the formula: *n* = (Z^2^ pq/d^2^) ^*^1.2. Where, *Z* = standard normal deviate, considered to be 1.96 at 95% confidence interval; *p* = expected proportions of health care utilization (*p* = 0.68) from a previous study (8); *q* = 1–p; *d* = 5% maximum allowable error; and with design effect 1.2. Therefore, the total sample size for this study was calculated to be 401. We used multistage, stratified, clustered sampling to obtain a sample representative of the municipality. The sampling occurred in following four steps:

Stage 1: Local wards served as the primary sampling units (PSUs). There are 33 wards in the study area. Census 2011 and information from District Statistics Office, Pokhara were used to generate the sampling frame and their corresponding populations. Of the total 33 wards in the Pokhara Lekhnath municipality, 17 wards were selected by simple random techniques using the “Decision analysis STATS version3” software. For each ward, the required sample size was estimated based on probability proportional to the ward size. The ward size was defined as the number of residential households in the ward.

Stage 2: Households within each ward were selected. At first, the center of the catchment area was reached, and the first household was selected by spinning a bottle or pencil. A bottle or pencil was spun, and the direction shown by the mouth of bottle or tip of the pencil was chosen for data collection. If the selected household did not have any eligible respondents, the “nearest door” rule was used, i.e., visit the household whose front door was closest to the door of the household.

Stage 3: Individuals were chosen to participate in the study from a list of all eligible persons residing in the selected households. One eligible participant was surveyed from each household. If more than one eligible participant was present in the household, the eldest participant was interviewed. There were no refusals.

### Data Collection and Variables

Data were collected through face to face interviews using a structured interview schedule. The survey tool was adapted from the Study on Global Aging and Adult Health (SAGE)'s questions on “Health Care Utilization.” Tools were developed in the English language and translated into the Nepali language and were then back-translated into English. The tool was pretested among 40 older adults in the Rambazar, a ward that was not selected during the random selection of the study PSUs. Following pretest, the revised versions of the tool were very similar to their original forms, with major differences involving changes in item wording, grammar, spellings, and removal of duplicate questions.

### Dependent Variables

#### Health Care Utilization

Data on prevalent comorbidities over the past year were self-reported by the participants. Participants were asked further questions regarding healthcare sought such as health care accessed, type of health facilities visited, reason for visit, mode of transportation, number of visits, and any overnight stay.

#### Health Care Expenditure

Participants were asked about their direct expenses on health care such as expenditure on the registration fee, emergency cost, medicine cost, laboratory fee, surgical cost, consultation fee, and accessories cost. Similarly, they were also asked about the indirect expenses on transportation cost and accommodation. All these expenses were self-reported by participants, with reference to their most recent health service utilization, in Nepali rupees which were converted to US dollars (1$ = 100 Nepali rupees) during data analysis. The total health care expenses were calculated as the sum of participant's direct and indirect expenses incurred during their most recent health care service utilization. Additionally, the source of income for meeting the health care expenses and their individual ability or reliance on others to meet the cost of expenses were asked.

### Explanatory Variables

#### Patient Satisfaction With Health Service

Participants were also asked to express their level of satisfaction (satisfied, neutral, and unsatisfied) regarding different aspects of health facilities and services such as waiting time, availability of staff and medications, attitude, politeness, and communication skills of health workers, and treatment or consultation time allocated.

#### Health Insurance

Participants' health insurance status, type, premium amount and services covered by health insurance, and their satisfaction with the insurance coverage were also assessed.

#### Socio-Demographic Variables

Socio-demographic variables included age, sex, ethnicity, religion, marital status, education, family type and size, the primary source of income, and the annual household income, all by self-report. The self-reported age was verified with DOB from a legal document, most often a citizenship document. For ethnicity, the Nepal Health Management Information System's classification was used; related categories were combined to obtain two ethnic groups: privileged included Upper Castes, and underprivileged included Janjatis, Dalits, and minorities. Educational status was categorized into two groups: illiterate, and literate.

International Wealth Index (IWI), asset-based wealth indices developed to measure welfare in developing countries, was used to quantify the economic situation of households (22). A household's ranking on IWI indicates to what extent the household possesses a basic set of assets, valued highly by people across the globe. Participants were asked to answer (yes/no) if they possessed a TV, refrigerator, phone, car, bicycle, cheap utensil, expensive utensil, and access to electricity (22). The quality of floor material, toilet facility, water supply (low, middle, high quality) and the number of rooms for sleeping (0 or 1, 2, 3 or more) were asked. The household's total IWI score (range: 0 to 100 with a higher score indicating higher housing quality) was calculated as the sum of the weighted asset value of these 12 assets (22). Then, the percentiles of the IWI score were calculated to indicate the household poverty level.

### Data Processing and Statistical Analyses

Data management and analyses were done in EpiData 3.2 and IBM SPSS16 (SPSS Inc. Chicago IL, USA), respectively. Values for numerical variables are expressed as the mean ± standard deviation (*SD*) and for categorical variables, as frequency (percentage). Differences in mean and frequency distributions between the groups were assessed using independent *t*-tests or Wilcoxon-Mann-Whitney test as applicable and Pearson's chi-square (χ^2^) tests, respectively. The predictors of health care utilization were assessed in binary logistic regression models. Coefficients were adjusted for age, sex, marital status, and family type. The histogram and Shapiro-Wilk's test (*p* < 0.001) suggested that health care expenditure was non-normally distributed. Therefore, out of pocket health expenditure is expressed in terms of median and interquartile range (IQR). The group differences in out of pocket health expenditure was assessed by Wilcoxon-Mann-Whitney test and Kruskal Wallis test. For all statistical tests, two-tailed *p*-values < 0.05 were considered statistically significant.

## Results

### Socio-Demographic Characteristics of Participants

The mean (±*SD*) age of the participants was 70.2 (±8.0) years. The majority of the participants were female (55.4%), privileged ethnicity (77.1%), married (55.1%), illiterate (57.6%), and lived in a joint family (80.5%). The mean (±*SD*) family size was 5.1 ± 2.4 and the median (IQR) annual household income was 5,000 (3,000–7,200), respectively (Table 1). Participants who did not visit a health facility in the previous year were more likely to be from an underprivileged ethnicity (*p* = 0.017), without a partner (*p* = 0.034), and living in a joint family (*p* = 0.020), and with a lower annual household income (*p* = 0.028) (Table 1).

**Table 1:** Socio demographic characteristics of participants by health service utilization (*n* = 401).

**Characteristics**	**Total (*n* = 401)**	**Health service utilization**		***p*-value**
		**Visited (*n* = 280)**	**Not visited (*n* = 121)**	
	***n* (%)**	***n* (%)**	***n* (%)**	
**Age**, mean ±*SD*	70.2 ± 8.0	70.1 ± 7.9	70.4 ± 8.2	0.793^a^
**Sex**				0.664
Male	179 (44.6)	123 (43.9)	56 (46.3)	
Female	222 (55.4)	157 (56.1)	65 (53.7)	
**Ethnicity**				**0.017**
Privileged	309 (77.1)	225 (80.4)	84 (69.4)	
Underprivileged	92 (22.9)	55 (19.6)	37 (30.6)	
**Marital status**				**0.034**
Married	221 (55.1)	164 (58.6)	57 (47.1)	
Without Partner	180 (44.9)	116 (41.4)	64 (52.9)	
**Family type**				**0.020**
Nuclear	78 (19.5)	46 (16.4)	32 (26.4)	
Joint	323 (80.5)	234 (83.6)	89 (73.6)	
**Family size**, Mean ±*SD*	5.1 ± 2.4	5.1 ± 2.4	4.9 ± 2.5	0.414^a^
**Education**				0.166
Literate	170 (42.4)	125 (44.6)	45 (37.2)	
Illiterate	231 (57.6)	155 (55.4)	76 (62.8)	
**Annual household income**, median (IQR)	5,000 (3,000–7,200)	5,000 (3,000–7,750)	4,400 (2,000–7,200)	**0.028**^**b**^
**Source of income**				**<0.001**
Business	118 (29.6)	88 (31.4)	30 (24.8)	
^1^Rent	34 (8.6)	24 (8.6)	10 (8.3)	
Nepal Government	34 (8.6)	27 (9.6)	7 (5.8)	
Pension from Abroad	65 (16.2)	52 (18.6)	13 (10.7)	
Remittance	72 (18.0)	51 (18.2)	21 (17.4)	
Other	78 (19.0)	38 (13.6)	40 (33.1)	
**International wealth index**, median (IQR)	88.8 (68.8–100.0)	88.8 (73.2–100.0)	88.8 (62.6–95.4)	0.234^b^

1 *Rent money collected from leasing house*.

a *P-value from independent t-test*.

b *p-value from Wilcoxon-Mann-Whitney test; all others are from chi-square test. Significant p-values are bolded*.

*SD, Standard deviation; IQR, Interquartile range*.

### Health Problems Among Participants

**Figure 1:**
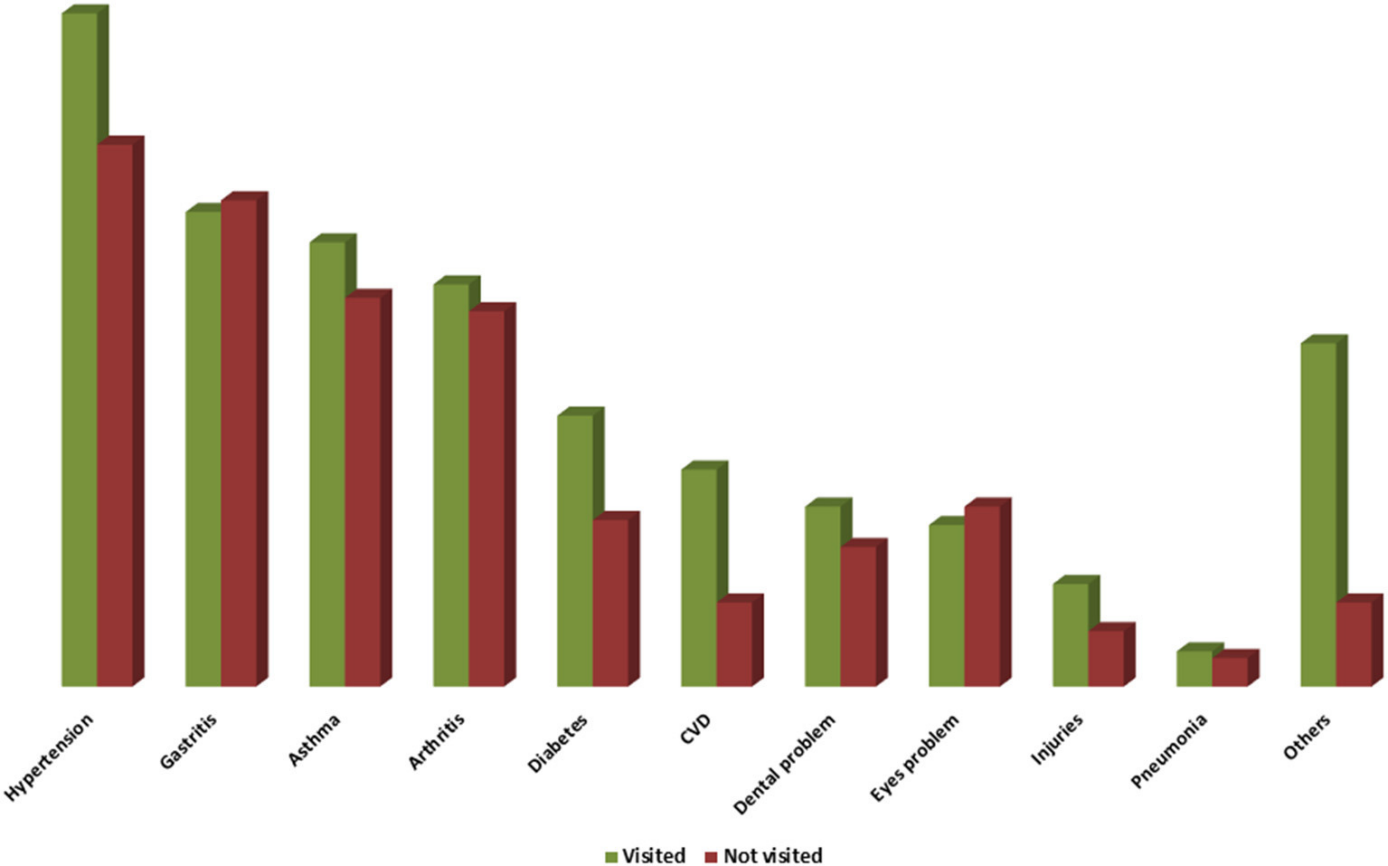
Health Problems among Study Participants. Others include Piles, urinary problem, uterine prolapse.

Hypertension (37.7%), gastritis (28.4%), asthma (25.4%), and arthritis (23.4%) were the major health problems reported among the participants in past year (Figure 1). The mean frequency of health problems in the past year was 2.8 ±1.3 (range 1–10).

### Health Care Utilization by the Participants

All of the study participants had some type of health problem in the last 12 months, but only 70% visited a health facility (Table 2). The utilization of outpatient and inpatient health care services were 87.5 and 14.6% respectively (not shown in table). The use of private health facilities (56.4%) was high compared to the use of government health facilities (35.7%). The main reason for a health facility visit was for the specific health problem encountered. Only 23% visited a health facility for a regular checkup. Public bus (47%) was the most common mode of transport for a health facility visit, and often the participants were accompanied by their children (49.3%) to the health facility. Only one-fifth of the participants had to stay overnight at the hospital, generally for 3 days (51.8%). Most (89.3%) of the participants were satisfied with the services received at the health facility. More than half (56%) of the study participants were not aware of the recently launched health insurance, and less than one-third of those aware of the insurance scheme had received insurance from the government source. Most of the participants (90.6%) who had insurance were satisfied with their insurance scheme (Table 2).

**Table 2:** Health services use indicators.

**Variables**	**Frequency (*n* = 401)**	**Percent**
**Visited health facility in past one year**		
Yes	280	69.8
No	121	30.2
^**a**^**Type of health facility visited**		
Government hospital	100	35.7
Private hospital	158	56.4
Health post	6	2.1
Community hospital	7	2.5
Army camp	4	1.4
Ayurvedic	5	1.8
^**a**^**Frequency of visit**		
Once	38	13.6
Twice	124	44.3
Thrice	60	21.4
More than 5 times	58	20.7
^**a**^**Reason for visit**		
Regular check up	65	23.2
Health problem	215	76.8
^***a***^**Distance from home**		
5 Km or less	265	96.3
More than 5 km	15	3.7
^**a**^**Means of transportation**		
By walking	18	6.4
Taxi	53	18.9
Bus	131	46.8
Own vehicle	78	27.9
^**a**^**Companion for visit**		
Children	138	49.3
Self	57	20.4
Daughter in law	32	11.4
Grand children	29	10.4
Spouse	19	6.8
Others (Friends and brothers)	5	1.8
^**a**^ **Admitted in hospital in past one year**		
Yes	56	20.0
No	224	80.0
**Duration of overnight hospital stay**		
One day	4	7.2
3 days	29	51.8
15 days	19	33.9
>1 month	4	7.2
^**a**^**Satisfaction with the services received**		
Satisfied	250	89.3
Dissatisfied	30	10.7
**Awareness of health insurance**		
Yes	175	43.6
No	226	56.4
**Membership of health insurance**		
Yes	53	30.3
No	122	69.7
^**b**^**Type of health insurance**		
Government	46	86.8
Private	7	13.2
^**b**^**Insurance premium**		
$25 or less	36	67.9
$25–30	1	1.9
$30–50	12	22.6
$50–100	4	7.5
^**b**^**Satisfied with health insurance**		
Yes	48	90.6
No	5	9.4

a *Questions asked only to participant who visited a health facility in the past 1 year*.

b *Questions asked only to participant who has health insurance*.

### Factors Associated With Health Care Utilization

In the binary logistic regression models, adjusted for age, sex, and annual household income; participants' ethnicity, marital status, annual household income, CVD, knowledge of health insurance, and having comorbidities were significantly associated with health care utilization (Table 3). Compared to participants from an underprivileged ethnicity, those from a privileged ethnicity had higher odds of utilizing the health services (OR: 1.66, 95% CI: 1.00–2.75), but the statistical significance was borderline. Older adults living with a partner had 72% higher odds of utilizing the health services compared to elderly participants without a partner (OR: 1.72, 95% CI: 1.06–2.77). Likewise, compared to participants from households with annual incomes <$1,000, those from households with higher (1,000–3,000) annual incomes were more likely to utilize the health services (OR: 2.70, 95% CI: 1.20–6.09). Diagnosis of CVD in the past 12 months (OR: 2.70, 95% CI: 1.10–6.63), knowledge of social insurance (OR: 4.78, 95% CI: 2.84–8.05), and multiple chronic conditions (OR: 5.92, 95% CI: 1.72–20.40) were associated with higher odds of utilizing the health services (Table 3).

**Table 3:** Binary logistic regression for factors associated with health care services utilization.

**Variables**	**Unadjusted**		**Adjusted**	
	**OR**	**95% CI**	**OR**	**95% CI**
^**a**^**Age**	1.00	0.98–1.03	1.00	0.98–1.03
^**b**^**Sex** (Reference = Male)				
Female	0.91	0.59–1.40	0.89	0.57–1.37
**Ethnicity** (Reference = Underprivileged)				
Privileged	**1.80**	**1.11–2.93**	**1.66**	**1.00–2.75**
**Marital status** (Reference = Without Partner)				
Married	**1.59**	**1.03–2.44**	**1.72**	**1.06–2.77**
**Family type** (Reference = Joint)				
Nuclear	**0.55**	**0.33–0.91**	0.65	0.38–1.12
**Family size**	0.96	0.88–1.05	0.99	0.90–1.08
**Education** (Reference = Illiterate)				
Literate	1.36	0.88–2.11	1.38	0.81–2.38
^**c**^**Annual household income** (Reference = Less than 1,000)				
1,000–3,000	**3.55**	**1.76–7.17**	**2.70**	**1.20–6.09**
More than 3,000	1.11	0.65–1.90	0.90	0.49–1.66
**CVD** (Reference = No CVD)	**2.83**	**1.16–6.90**	**2.70**	**1.10–6.63**
**Awareness of health insurance** (Reference = No)				
Yes	**4.73**	**2.86–7.84**	**4.78**	**2.84–8.05**
**Insurance status** (Reference = No)				
Yes	1.06	0.41–2.74	0.99	0.38–2.61
**Morbidity** (Reference = None)				
One	1.24	0.79–1.94	1.18	0.75–1.86
iMore than one	**5.99**	**1.75–20.53**	**5.92**	**1.72–20.40**

a *Adjusted for sex and annual household income*.

b *Adjusted for age and annual household income*.

c *Adjusted for age and sex. All others adjusted for age, sex, and annual household income. CVD, Cardio-vascular disease. Significant p-values are bolded*.

### Health Care Expenses

The average out of pocket health expenditure per visit was 41.3 (IQR = 0–125.3) (not shown in table). The major source of income for meeting the health care expenses was rent money collected from leasing a house (26.4%), pension (24.3%), and borrowing money (15%) (not shown in table). The out-of-pocket health expenditure was either provided by the participants themselves (42.9%), or they relied on their son and daughter-in-law to cover the expenses (47.5%) (not shown in table). Compared to privileged ethnic groups, the underprivileged group had significantly lower health expenditures (*p* = 0.004) (Table 4). Out-of-pocket health expenditures was higher among married participants (*p* = 0.009) and in higher income households (*p* = 0.005). Out-of-pocket health expenditures were significantly higher among participants attending private health facilities (*p* = <0.001) and those with a diagnosis of CVD (*p* = <0.001) and knowledge of social insurance (*p* = <0.001). The out-of-pocket health expenditure increased gradually with the increase in number of morbidities (*p* = <0.001) (Table 4).

**Table 4:** Out of pocket health expenditure (in US Dollar) during last recent visit.

**Variables**	**Out of pocket health expenditure ($)**	***P*-value**
	**Median cost ($) (Q1-Q3)**	
**Age, Years**		0.646^a^
60–69	41.0 (0.0, 100.0)	
70–79	49.0 (0.0, 131.0)	
>=80	32.9 (0.0, 125.5)	
**Sex**		0.611
Male	46.4 (0.0, 135.3)	
Female	40.0 (0.0, 104.0)	
**Ethnicity**		**0.004**
Privileged	45.5 (0.0, 133.3)	
Underprivileged	22.8 (0.0, 72.5)	
**Marital status**		**0.009**
Married	59.0 (0.0, 137.3)	
Without Partner	28.1 (0.0, 88.2)	
**Family type**		0.071
Nuclear	15.3 (0.0, 125.0)	
Joint	45.5 (0.0, 126.3)	
**Education**		0.103
Literate	52.1 (0.0, 135.5)	
Illiterate	36.3 (0.0, 113.3)	
**Annual household income $**		**0.005**^**a**^
Less than 1,000	0.0 (0.0, 48.5)	
1,000–3,000	51.6 (0.0, 135.5)	
More than 3,000	42.8 (0.0, 128.5)	
**Type of facility**		**<0.001**
Public	49.6 (20.3, 113.3)	
Private	97.3 (55.0, 215.3)	
**Morbidity**		
None	20.3 (0.0, 80.3)	**<0.001**^**a**^
One	45.3 (0.0, 135.5)	
More than One	119.1 (35.0, 292.0)	
**Cardio-vascular diseases**		**<0.001**
Yes	192.0 (38.3, 416.0)	
No	36.9 (0.0, 96.0)	
**Satisfaction with received health services**		0.117
Satisfied	80.3 (40.0, 174.3)	
Dissatisfied	54.5 (20.3, 125.0)	
**Knowledge of social insurance**		**<0.001**
Yes	66.5 (20.6, 158.8)	
No	16.3 (0.0, 80.3)	
**Insurance status**		0.272
Yes	94.3 (20.6, 187.0)	
No	64.0 (23.6, 128.5)	

a *P-value from Kruskal Wallis test; all others are from Wilcoxon-Mann-Whitney test. Significant p-values are bolded*.

## Discussions

Our study aimed to assess health care utilization among older adults in Pokhara-Lekhnath Municipality and found that a high percentage of the population 60 years and older reported using in- and out-patient services and incurred a high out-of-pocket health expenditure. Nevertheless, 30% of the participants did not visit a health facility which, when translated to a total population of 42,935 in the Pokhara-Lekhnath Municipality, is equivalent to 12,880 individuals (1). This finding is concerning because our study participants had at least one health problem in past 12 months and 30% still did not seek a health care service. It is possible that older adults in the general population and/or those free of any disease symptoms will have even less access to health care. Furthermore, our study was conducted in an urban setting, and the use of health care utilization may be worse in rural settings where sufficient services and manpower are not available.

There are limited studies from Nepal looking at health care utilization among the elderly population, and none have looked into out of pocket medical expenses in this population (8, 14). A previous study from Nepal also reported a similar proportion (68%) of older adults using health care services in the last 12 months (8). However, a separate study, conducted among older adults in urban Nepal, reported a higher proportion (85%) of health care service utilization (14). The higher rates of utilization in the latter study may be attributed to the urbanized study setting because health disparity in terms of urban-rural settings is almost universal.

For senior citizens, aged 70 years and above, the Government of Nepal provides free health checkups through public hospitals and health facilities and treatment subsidies for severe health conditions such as cancer, heart disease, uterus prolapse, and kidney disease. Despite this, private health facilities were more popular than the government health facilities in this study and previous studies from Nepal and other Asian countries (8, 23, 24). Private health facilities in Nepal, although less in number and limited to more developed parts of the country, are very popular as they provide higher quality health services, are easily accessible, offer less waiting time and provide the machines and technology that are needed to diagnose and treat a wide variety of diseases (14, 25). Utilization of private facilities is directly related to an individual's ability to cover the cost (26). Since Pokhara is one of the most expensive cities in Nepal, and a significant proportion of the local elderly residents are retired Gurkha army supported by pensions from abroad, this may explain people's choice to utilize private facilities even when the public health facilities provide free health care to older adults. However, high utilization of private health care can lead to an increase in out-of-pocket health expenditures (23).

Socioeconomic vulnerabilities are important determinants of health care utilization in developing countries (23, 27). Our findings of positive associations between health care utilization and privileged ethnicity (14), being married (8), a higher annual household income (28), and a knowledge of social insurance (14) are consistent with previous studies. In Nepal, the underprivileged ethnic groups such as the Dalit and disadvantaged Janjatis, almost all fall under the poverty line, and have limited access to health care and education (29). Socioeconomic status (SES) ensures affordability of daily needs and is highly associated with many health outcomes and access to health care (28, 30, 31). The precise mechanisms by which marriage confers health benefits are unclear, but studies show that married adults have better health and survival (32). The higher levels of healthcare utilization among married individuals can be attributed to better social, psychological, and financial support received from the spouse, as well as companionship for visiting the health facility and reminding the spouse of appointments (33).

Different health problems such as hypertension, gastritis, asthma, and arthritis are common among elderly participants in our study and others (6, 8, 19). During life transitions, various physical, mental and psycho-social changes accompany the phenomenon of aging (34). Biologically, aging is characterized by a gradual, lifelong accumulation of molecular and cellular degeneration that subsequently leads to a decrease in physiological functions, a compromised immune function, and an increased vulnerability to diseases (35, 36). Thus, with increasing age, people are more likely to experience multi-morbidities. One of the noteworthy findings in the study is that participants often went to a health facility to seek treatment for a specific health problem while the practice of regular health check-ups was low. Findings from previous studies support our results (8). Especially among older adult populations, it is recommended that people visit health care providers, for a well-care visit, at least once a year in order to prevent diseases and/or catch them at early onset (37).

Our findings of higher out-of-pocket health expenditures among privileged ethnic groups, married participants, those from higher income households, and attending private health facilities are supported by previous studies (38, 39). Ethnicity is an important social determinant of health; ethnic minorities are less likely to use medical services than other populations (39). Our findings may be explained by the fact that the underprivileged ethnic groups, who are the disadvantaged group with a lower SES, may have limited access as well as the financial resources to afford high out-of-pocket health expenses (38). The higher out-of-pocket health expenditures among married participants, although not clearly understood, may be due to the financial support received from the spouse, or it may be due to higher levels of healthcare utilization by the participants because of encouragement and companionship of spouse for visiting the health facility (33). A correlation between income or SES and several aspects of health expenditure is indisputable (38). Household income and/or SES provides a measure of an individual's affordability and subsequently reflects the expenditure made in health care. The higher out-of-pocket health expenditures among participants visiting private health facilities compared to public health facilities were expected given that the government of Nepal provides free primary health care to the senior citizens, but the private health facility charges a substantial amount for their services. Similarly, participants with a diagnosis of CVD had higher out-of-pocket health expenditures. The finding was not surprising given that CVD is **one** of the most costly diseases with high financial burden incurred for the diagnosis, treatment, and rehabilitation services (40).

### Strengths and Limitations of the Study

The large sample size and representative sample selection are strengths of this study. One limitation of this study is its cross-sectional design. The information on health status and health care utilization and expenditure is self-reported by the participants which may not reflect the true estimates of the parameters. However, self-reported information on health has been a widely accepted approach in practice, especially when available resources are limited (41). There may also be recall bias, especially for expenditure data, which may affect the results. Another important limitation of the study is that it does not take into account the duration of stay in inpatient care as high out-of-pocket expenditure incurs in coordination with duration of hospital stay. The generalizability may be limited to similar geographical urban areas because difficult geography, distance to health facility, and lack of transportation are the primary barriers to health care utilization in remote hilly and mountainous areas of the country.

Until now, there have been a limited number of studies uncovering the interaction between the aging process and health in the context of Nepal. Our study contributes to the comparability of information with a previous study from Nepal and among countries with the same demographic dynamics (14). The current estimates for health care utilization, the factors intervening in the utilization, and the household's incurred expenditures represent an advance and a platform for future research. Our research can be a reference for future research in Nepal. Future studies should estimate health spending by age groups within the elderly, and by ethnic variability.

## Conclusions

The older adult population in Nepal is increasing rapidly. Increasing access to health care is critical for achieving a higher quality of health among older adults. The public health system must develop effective strategies to attract this portion of society. High dependency on private health facilities, as noted in the study, will only lead toward high out-of-pocket health expenditures. The health benefits of regular health screenings must be disseminated among the elderly population. The role of preventive care services in timely diagnosis and treatment of disease and reducing the total health care expenditure by providing cost-effective treatment in early stages should be emphasized. Knowledge regarding free health services provided by the government of Nepal to the older adult population should be disseminated. Awareness programs targeting the underprivileged ethnic groups and poor households are recommended. Multiple strategies for sharing information of the recently introduced social health insurance may provide increased access to health care. Developing quality and affordable health care services for older adults to ensure equity in accessibility will be a major task for the public health system in Nepal.

## Ethics Statement

The Institutional Review Committee (IRC) at Pokhara University approved this study. Written permission was received from the Pokhara Lekhnath metropolitan office. Informed written consent was received from the respondents before initiating data collection. Participation was voluntary, and participants' identity was kept confidential.

## Author Contributions

SA and NS: conceived and designed the study and facilitated data collection in the field; SG: analyzed the data; SA, SG, and EJ: drafted the manuscript; SA, SG, EJ, and NS: critical revision of the manuscript; SA, SG, EJ, and NS: approval of the final version of the manuscript.

### Conflict of Interest Statement

The authors declare that the research was conducted in the absence of any commercial or financial relationships that could be construed as a potential conflict of interest.

## References

[1] Central Bureau of Statistics National Population and Housing Census 2011. National Report. Government of Nepal, National Planning Commission Secretariat. Kathmandu (2012).

[2] Nepal Law Commission Senior Citizens Act 2063. Kathmandu (2006). Available online at: https://bit.ly/2wFKlja

[3] Geriatric Center Nepal Status Report on Elderly People (60+) in Nepal on Health, Nutrition and Social Status Focusing on Research Needs. Kathmandu Geriatric Center Nepal and Government of Nepal, Ministry of Health and Population (2011).

[4] Wolff JL Starfield B Anderson G. Prevalence, expenditures, and complications of multiple chronic conditions in the elderly. Arch Intern Med. (2002) 162:2269–76. 10.1001/archinte.162.20.2269 12418941

[5] Barnett K Mercer SW Norbury M Watt G Wyke S Guthrie B. Epidemiology of multimorbidity and implications for health care, research, and medical education: a cross-sectional study. Lancet (2012) 380:37–43. 10.1016/S0140-6736(12)60240-2 22579043

[6] Australian Bureau of Statistics Australian Health Survey: First Results 2011-12. Canberra (2012). Available online at: https://bit.ly/2IocufK

[7] World Health Organization Preventing chronic diseases: a vital investment. Geneva: WHO, 2005. Revista Baiana de Saúde Pública (2016).

[8] Sanjel S Mudbhari N Risal A Khanal K. The utilization of health care services and their determinants among the elderly population of Dhulikhel Municipality. Kathmandu Univ Med J. (2012) 10:24–9. 10.3126/kumj.v10i1.6911 22971859

[9] Hoffman C Rice D Sung HY. Persons with chronic conditions. Their prevalence and costs. JAMA (1996) 276:1473–9. 8903258

[10] Xiaolong Z Qiong C Jin W Yun L Determinants of medical and health care expenditure growth for urban residents in China: a systematic review article. Iran J Public Health (2014) 43:1597–1604. 26171351 PMC4499080

[11] Shrestha L. Geriatric health in Nepal: concerns and experience. Nepal Med Coll J. (2012) 15:144–8. 24696938

[12] Farrow FL Overutilization and underutilization of preventive services in elderly populations: a conundrum. marq. Elder Adviser (2010) 12:103.

[13] Lipitz-Snyderman A Bach PB Overuse of health care services: when less is more…more or less. JAMA Intern Med. (2013) 173:1277–8. 10.1001/jamainternmed.2013.6181 23712254 PMC3805025

[14] Gurung L Paudel G Yadav U Health service utilization by elderly population in urban nepal: a cross-sectional study. J Manmohan Memor Inst Health Sci. (2016) 2:27–36. 10.3126/jmmihs.v2i0.15794

[15] Kibria A Mancher M McCoy MA Graham RP Garber AM Newhouse JP Variation in Health Care Spending: Target Decision Making, Not Geography (2013). Washington, DC: National Academies Press. 24851301

[16] Zayas CE He Z Yuan J Maldonado-Molina M Hogan W Modave F. Examining healthcare utilization patterns of elderly middle-aged adults in the United States. Proc Int Fla AI Res Soc Conf. (2016) 2016:361–6. 27430035 PMC4946167

[17] World Bank Group. World Bank Data on Nepal (2016). Available online at: https://data.worldbank.org/country/Nepal

[18] Hotchkiss DR Rous JJ Karmacharya K Sangraula P. Household health expenditures in Nepal: implications for health care financing reform. Health Policy Plan (1998) 13:371–83. 10346029 10.1093/heapol/13.4.371

[19] Adhikari D Rijal DP Factors affecting health seeking behavior of senior citizens of Dharan. J Nobel Med Coll. (2014) 3:50–7. 10.3126/jonmc.v3i1.10055

[20] Shrestha MV Paudel L Pant S Neupane S Manandhar N Health seeking behavior among women in Bhimtar, Sindhupalchowk district of Nepal. Int J Commun Med Public Health (2017) 4:1854–7. 10.18203/2394-6040.ijcmph20172144

[21] Bisht PS Pathak RS Subedi G Shakya DV Gautam KM Health and Social Care Needs Assessment of Elderly: The Context of Piloting Service Developments and Care of Elderly in Pharping (2012). Kathmandu: Central Department of Population Studies, Tribhuvan University.

[22] Smits J Steendijk R The international wealth index (IWI). Soc Indicat Res. (2015) 122:65–85. 10.1007/s11205-014-0683-x

[23] van Doorslaer E O'Donnell O Rannan-Eliya RP Somanathan A Adhikari SR Garg CC. Effect of payments for health care on poverty estimates in 11 countries in Asia: an analysis of household survey data. Lancet (2006) 368:1357–64. 10.1016/S0140-6736(06)69560-3 17046468

[24] Xu K Evans DB Kawabata K Zeramdini R Klavus J Murray CJ. Household catastrophic health expenditure: a multicountry analysis. Lancet (2003) 362:111–7. 10.1016/S0140-6736(03)13861-5 12867110

[25] Mackintosh M Channon A Karan A Selvaraj S Cavagnero E Zhao H. What is the private sector? Understanding private provision in the health systems of low-income and middle-income countries. Lancet (2016) 388:596–605. 10.1016/S0140-6736(16)00342-1 27358253

[26] Saito E Gilmour S Yoneoka D Gautam GS Rahman MM Shrestha PK. Inequality and inequity in healthcare utilization in urban Nepal: a cross-sectional observational study. Health Policy Plan (2016) 31:817–24. 10.1093/heapol/czv137 26856362 PMC4977425

[27] Kowal P Chatterji S Naidoo N Biritwum R Fan W Lopez Ridaura R. Data resource profile: the World Health Organization Study on global AGEing and adult health (SAGE). Int J Epidemiol. (2012) 41:1639–49. 10.1093/ije/dys210 23283715 PMC3535754

[28] DeNavas-Walt C Income, Poverty, and Health Insurance Coverage in the United States (2005) (2010). Washington, DC: DIANE Publishing.

[29] Bhandari S Sayami JT Thapa P Sayami M Kandel BP Banjara MR. Dietary intake patterns and nutritional status of women of reproductive age in Nepal: findings from a health survey. Arch Public Health (2016) 74:2. 10.1186/s13690-016-0114-3 26823976 PMC4730652

[30] Adler NE Newman K. Socioeconomic disparities in health: pathways and policies. Health Aff. (2002) 21:60–76. 10.1377/hlthaff.21.2.60 11900187

[31] Braveman P Egerter S Barclay C Issue Brief Series: Exploring the Social Determinants of Health: Income, Wealth, and Health. (2011) Princeton, NJ: Robert Wood Johnson Foundation.

[32] Rendall MS Weden MM Favreault MM Waldron H. The protective effect of marriage for survival: a review and update. Demography (2011) 48:481–506. 10.1007/s13524-011-0032-5 21526396

[33] DiMatteo MR. Social support and patient adherence to medical treatment: a meta-analysis. Health Psychol. (2004) 23:207–18. 10.1037/0278-6133.23.2.207 15008666

[34] World Health Organization World Report on Ageing and Health (2015). Luxembourg: World Health Organization.

[35] Castelo-Branco C Soveral I. The immune system and aging: a review. Gynecol Endocrinol. (2014) 30:16–22. 10.3109/09513590.2013.852531 24219599

[36] Cruz-Jentoft AJ Baeyens JP Bauer JM Boirie Y Cederholm T Landi F. Sarcopenia: European consensus on definition and diagnosis: report of the European working group on sarcopenia in older people. Age Ageing (2010) 39:412–23. 10.1093/ageing/afq034 20392703 PMC2886201

[37] Dunn J Adult Well-Care Visits, Screenings, and Immunizations (2018). Available online at: https://k-p.li/2Gjqshl

[38] Tur-Sinai A Magnezi R Grinvald-Fogel H. Assessing the determinants of healthcare expenditures in single-person households. Israel J Health Policy Res. (2018) 7:48. 10.1186/s13584-018-0246-8 30318017 PMC6191996

[39] Scheppers E Van Dongen E Dekker J Geertzen J Dekker J. Potential barriers to the use of health services among ethnic minorities: a review. Family Pract. (2006) 23:325–48. 10.1093/fampra/cmi113 16476700

[40] Tarride JE Lim M DesMeules M Luo W Burke N O'Reilly D. A review of the cost of cardiovascular disease. Can J Cardiol. (2009) 25:e195–202. 19536390 10.1016/s0828-282x(09)70098-4 PMC2722492

[41] He W Muenchrath MN Kowal PR Shades of Gray: A Cross-Country Study of Health and Well-Being of the Older Populations in SAGE Countries 2007-2010: US Department of Commerce. Economics and Statistics Administration (2012). US Census Bureau.

